# Local Stressors Reduce Coral Resilience to Bleaching

**DOI:** 10.1371/journal.pone.0006324

**Published:** 2009-07-22

**Authors:** Jessica E. Carilli, Richard D. Norris, Bryan A. Black, Sheila M. Walsh, Melanie McField

**Affiliations:** 1 Scripps Institution of Oceanography, University of California San Diego, La Jolla, California, United States of America; 2 Hatfield Marine Science Center, Oregon State University, Newport, Oregon, United States of America; 3 Smithsonian Institution, Belize City, Belize; NOAA/NMFS/SWFSC, United States of America

## Abstract

Coral bleaching, during which corals lose their symbiotic dinoflagellates, typically corresponds with periods of intense heat stress, and appears to be increasing in frequency and geographic extent as the climate warms. A fundamental question in coral reef ecology is whether chronic local stress reduces coral resistance and resilience from episodic stress such as bleaching, or alternatively promotes acclimatization, potentially increasing resistance and resilience. Here we show that following a major bleaching event, *Montastraea faveolata* coral growth rates at sites with higher local anthropogenic stressors remained suppressed for at least 8 years, while coral growth rates at sites with lower stress recovered in 2–3 years. Instead of promoting acclimatization, our data indicate that background stress reduces coral fitness and resilience to episodic events. We also suggest that reducing chronic stress through local coral reef management efforts may increase coral resilience to global climate change.

## Introduction

**Figure 1:**
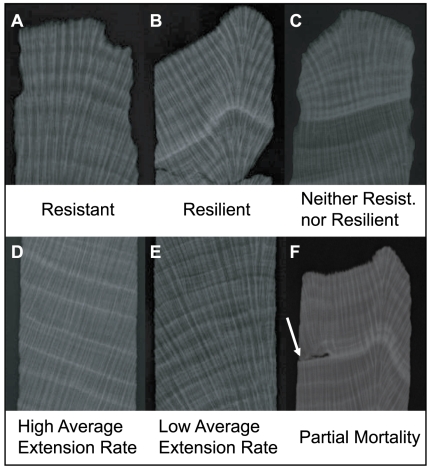
X-radiographs of various coral cores showing the different types of growth behavior discussed. (A) Coral without the 1998 growth suppression, indicating resistance to bleaching in 1998. (B) Coral with the 1998 growth suppression, recognized by the bright high-density band, but with a quick return to pre-1998 extension rates, indicating resilience after bleaching. (C) Coral with the 1998 growth suppression and continuing depressed extension rates after 1998, indicating a lack of both resistance and resilience to bleaching. (D) A coral with relatively high average extension rate. (E) A coral with relatively low average extension rate. (F) A coral with a partial mortality scar on the left (noted by white arrow), coincident with the 1998 growth anomaly.

**Figure 2:**
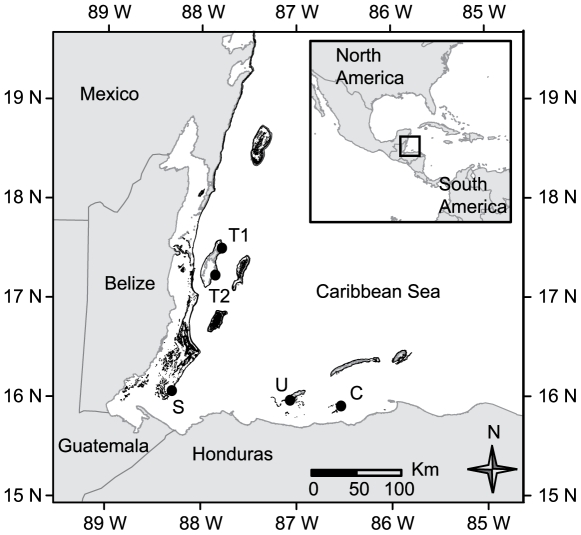
Map of the Mesoamerican Reef with locations of coral collections as black circles. Dark grey denotes coral, light grey denotes land areas. T1, T2 = Turneffe Atoll (4 cores from T1, 13 from T2), S = Sapodilla Cayes (44 cores), U = Utila (17 cores), C = Cayos Cochinos (14 cores).

Ecological studies have demonstrated that stressors currently affecting coral reefs include, among others, coral diseases [1], over-fishing [2], and a combination of pollution and sedimentation from coastal development [3]. These chronic stressors are often associated with the gradual loss of coral cover and overgrowth by fleshy algae. However, abrupt and severe episodic events, such as coral bleaching, may also be responsible for coral reef degradation [4]. An outstanding issue is whether the combination of multiple stressors reduces coral resistance or resilience to episodic events such as bleaching [5]–[7], or alternatively whether acclimatization to stressful conditions can increase coral resistance—the ability of corals to withstand future stress [8], [9]. Bleaching is a generalized term for the loss of symbiotic dinoflagellate zooxanthellae or their pigments in scleractinian corals and is typically associated with sustained, unusually warm water temperatures [10]. Several studies have found that bleaching reduces skeletal growth in corals [11]–[13]. Ocean acidification may also reduce the ability of corals to calcify as normal; a recent study of corals from the Great Barrier Reef attributes a 14.2% decrease in calcification since 1990 to a combination of acidification and warming [14]. Here we define resistance as an individual coral's ability to continue normal skeletal growth even under stress (whether chronic or episodic), and resilience as a coral's ability to recover to normal growth rates after a stressful event (Fig. 1a–c). To test our hypothesis that chronic local stress reduces coral resistance and resilience to bleaching, we focus on coral growth before and after the 1998 mass-bleaching event [15] from four sites on the Mesoamerican Reef (Fig. 2, Table 1) with relatively high and low chronic local stress.

**Table 1:** Coral core collection site locations.

Site	Dive Site Name	Coordinates	Total Cores	1998 Stress Band	Partial Mortality in 1998	Previous Stress Bands
Turneffe 1	Dog Flea Caye	17°29′59″N, 87°45′30″W	17	71% (12)	6% (1)	2
Turneffe 2	Harry Jones	17°18′25″N, 87°48′04″W				
Sapodilla	Frank's Caye, NE buoy	16°07′45″N, 88°14′59″W	44	100% (44)	16% (7)	0
Utila	Diamond Caye	16°03′52″N, 86°57′30″W	17	100% (17)	12% (2)	1
Cayos Cochinos	Pelican Point, Peli 2	15°58′41″N, 86°29′06″W	14	100% (14)	21% (3)	0
		**Total**	**92**	**95% (87)**	**14% (13)**	**3**

Location names with dive site name or nearby caye, coordinates, number of cores from each site, along with growth anomalies in 1998 and earlier. Table lists the total number of cores which were drilled and slabbed along the growth axis, the percentage and number of these that have dense stress bands associated with the 1998 event, the percentage and number that contained a partial mortality scar, and any previous individual stress bands.

## Methods

The level of local stress at each site was calculated using the methodology of Halpern et al. (2008) [16] to estimate a cumulative impact index based on weighted, log-transformed, re-scaled data representing (1) sedimentation (2) nutrient input (3) local human population size adjacent to our sites and (4) a relative measure of fishing pressure based on fish abundance surveys [17, supplementary information Text S1] (Table 2). Our calculated local impact scores for the Mesoamerican reef sites have the same ranking as the “Halpern Index” that we calculated as the average of 4 cells representing our sites from the Halpern et al. (2008) [16] global impact map. We also compared our results with the integrated reef health index at our sites from the Healthy Reefs Report Card [17] (Table 2). According to all three indices of local stressors and reef health, our sites in the Sapodilla Cayes and Utila experience the highest levels of local stress, while sites in Cayos Cochinos and Turneffe Atoll experience lower levels.

**Table 2:** Anthropogenic stress scores.

Site	Sedimentation	Nutrients	Human Population	Fishing Pressure	Stress Index	Halpern Index	Healthy Reefs Index*
Sapodilla	*1.60*	*1.80*	*0.00*	*0.00*	***3.40***	13.35	2.10
Utila	*0.91*	*0.56*	*2.30*	*−2.08*	***1.70***	13.27	2.49
Cayos Cochinos	*1.37*	*0.89*	*1.00*	*−2.41*	***0.84***	11.06	2.98
Turneffe	*0.00*	*0.00*	*0.31*	*−0.11*	***0.20***	9.80	2.59

Four measures of local stress at each site: sedimentation, nutrients, human population and fishing pressure used to calculate our cumulative “Stress Index” (sources in supplemental information). A comparison with the Halpern et al. (2008) [15] and Healthy Reefs [16] indices is also shown, though note that only the relative rankings are comparable. Sapodilla and Utila experience higher local impacts and lower reef health. *Note that while low Stress Index and Halpern Index scores indicate fewer local stressors, a low Healthy Reefs Index score indicates worse reef health.

**Figure 3:**
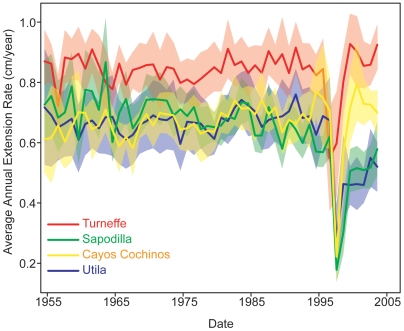
Bootstrapped means (solid lines) and 95% confidence intervals (shading) for extension rates after 1955. Extension rates at Sapodilla and Utila remain suppressed after the 1998 bleaching event (p<0.001).

We collected a total of 92 coral cores from *Montastraea faveolata*, the dominant reef-builder on the fore reef [18] between 2.5–13 m depth in spur and groove habitat (Table 1). Permission to collect and export coral samples was granted by the Belize Fisheries Department and the Secretaria de Agricultura y Ganadería, Honduras. Coral skeletal growth rates were measured based on annual density bands in the skeleton [19], supplementary information Text S1. Coral extension, average density, and calcification (the product of extension and density) were analyzed from digital x-rays using the program CoralXDS [20] for each high-density, low-density annual couplet, though in this paper we focus on extension rates (cm/year). To ensure all growth-increment chronologies were annually resolved, we made novel use of tree-ring techniques, including “crossdating” of annual extension rate (growth-increment width). Average extension rates and 95% confidence intervals were calculated using 10,000 bootstrapped samples (Fig. 3). Differences between sites were compared using pairwise permutation tests on extension rates between 1950–1997. Edinger et al. (2000) [21] found that comparing absolute extension rates between sites is not necessarily representative of overall reef health. Indeed, Turneffe Atoll, one of the less impacted sites, has significantly (p<0.001) higher extension rates than the other three sites before 1998, but this site also lost significant coral cover due to the 1998 bleaching event and hurricane Mitch (Fig. S1). Therefore, here we focus on resistance to and recovery after bleaching in 1998 instead of absolute differences between sites. Recovery to normal mean extension rates after 1998 were estimated, while controlling for the year, by fitting the generalized linear model (GLM): (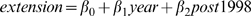) (Table S1). Extension rates from the years 1950 to 1997 (post1998 = 0) were compared to rates from 2002 to 2006 (post1998 = 1) to avoid the years 1998–2001 during which all sites had severely depressed growth.

## Results and Discussion

At all four sites, nearly every coral displays a visually prominent stress band in 1998 [11] (Fig. 1b), indicated by an increase in skeletal density and decrease in extension and calcification rates more than four standard deviations outside the mean chronology. Indeed, 95% of coral cores show a marked reduction in extension rates that persists for two years or more following the 1998 event (Fig. 3, Table 1). Additional rare stress bands (denser than the long-term mean by at least 1.5 standard deviations) are seen in three individual coral cores in 1950, 1965 and 1995. However, no other coral cores displayed significant changes in growth during these years, nor did we observe any partial mortality scars before 1998.

Although the 1998 bleaching event affected the entire Mesoamerican reef system, there are spatial differences in its intensity revealed in our coral cores. We compared the number of coral cores with partial mortality scars (Fig. 1f) and 1998 stress banding between sites using a permutation test. Turneffe Atoll, the site with the lowest level of local stress, is also the only site without ubiquitous stress banding (p<0.05) (Fig. 1a), and has the lowest, but non-significant, frequency of partial mortality scars (Table 2). The lack of stress banding in some coral cores at Turneffe Atoll suggests that at least some of those corals may have resisted bleaching. By comparison, the universal occurrence of stress banding at all other sites supports the hypothesis that high chronic stress decreases coral resistance to bleaching.

In contrast to the modest variability in resistance to the 1998 bleaching event, there are large differences in the resilience of corals between sites. At Sapodilla and Utila where stress indices are high, coral growth still did not recover completely by the time of collection more than eight years following the 1998 bleaching event, even controlling for long-term decreasing trends at these sites (GLM: Sapodilla, F_(2,50)_ = 20.70, p<0.001, R^2^ = 0.45; Utila, F_(2,50)_ = 18.26, p<0.001, R^2^ = 0.42, Table S1). Such a long recovery period is unprecedented in the literature, with most studies reporting growth suppression due to bleaching on the order of one year [11]–[13] and the longest growth suppression reported for four years [22]. In comparison, corals from lower-stress sites (Turneffe and Cayos Cochinos) recovered to pre-bleaching extension rates in about three years (Fig. 3, Table S1). However, at sites with high local stress, corals have been unable to recover to pre-disturbance growth rates.

The between-site differences in both the level of impact of the 1998 bleaching event and the subsequent recovery time are not easily explained by between-site differences in the strength of the 1998 bleaching event. We tested the hypothesis that our sites experienced differences in heat stress in 1998 by calculating the degree-heating-weeks (DHW) [23] for our four study areas from 7-day composite night time sea surface temperature data [supplementary information Table S1]. Our findings indicate that during 1998, heat stress was higher at Cayos Cochinos and the Sapodilla Cayes (6.84 and 5.54 maximum DHW, respectively), compared to Utila and Turneffe Atoll (3.34 and 2.27 maximum DHW, respectively). However, while lower temperature stress may help explain the lower stress banding at Turneffe it cannot explain the lack of resilience at Utila or the higher resilience in Cayos Cochinos (which experienced the highest heat stress).

We also examined the possibility that a hurricane strike may have had different effects across the Mesoamerican Reef. Category 5 hurricane Mitch (October 21–29, 1998) produced extreme runoff over most of the southern portion of the Mesoamerican Reef and reduced water clarity for several weeks [24]. However, we have found no geochemical signature associated with runoff from Mitch, even in coral cores analyzed at extremely high resolution using laser ablation [25]. The lack of signal indicates the corals stopped calcifying due to the bleaching event (August 1998) prior to the hurricane. While poor water quality could explain the subsequent low resiliency of corals at our southern sites, it is notable that corals at the most southerly site, Cayos Cochinos, recover to pre-1998 growth rates just as rapidly as corals at Turneffe where the runoff impact of the hurricane was low. In addition, earlier hurricane strikes have left almost no record in our coral growth rate data suggesting that their overall impact has been low on *Montastraea faveolata*.

We conclude that the large differences in chronic stress between our sites are responsible for differences in coral resilience following exposure to the 1998 bleaching event. To date the 1998 bleaching event remains the most significant bleaching event recorded on the Mesoamerican reef, as the 2005 event was significantly less severe than in other parts of the Caribbean [26]. Our data do not support the hypothesis that exposure to stress might help coral colonies acclimatize and therefore resist bleaching. Instead, it is clear that coral colonies experiencing higher local stress before 1998 were more severely affected by bleaching and recovered more slowly than those exposed to lower chronic stress. Possibly, the acclimatization hypothesis is only applicable for the same stressor or for lower levels of stress than Sapodilla and Utila experience, and the multi-species coral community may exhibit acclimatization patterns different from the individual coral colony response measured in this study. For example, repetitive bleaching may increase a coral's ability to withstand future heat stress [8], [9], but other local stressors such as increased sedimentation may depress a coral's energy reserves [27], making it less likely to survive or recover from a bleaching event [28]. Even if acclimatization can occur in some cases, the differential responses of *M. faveolata* across various stress regimes indicate that local conservation efforts that reduce stress, such as reducing runoff by replanting mangroves at the coast or protecting an area from overfishing, could have significant impacts on the ability of corals to withstand the effects of climate change. Future research could investigate whether this interaction between local and global stressors extends to other coral species.

## Supporting Information

Text S1 Detailed Materials and Methods as well as a supplementary table of the outcome of our statistical test for growth rate recovery after bleaching, and a supplemental figure showing coral cover before and after 1998.
(0.06 MB DOC)

Figure S1 Supplemental figure of coral cover before and after 1998.
(3.79 MB TIF)

Table S1 Recovery of extension rates after 1998.
(0.03 MB DOC)
