# Supplementary material for: Local Stressors Reduce Coral Resilience to Bleaching
Source: PLoS One. 2009 Jul 22;4(7):e6324. doi: 10.1371/journal.pone.0006324 (PMC2708352; doi:10.1371/journal.pone.0006324)
Supplement: Text S1 — Detailed Materials and Methods as well as a supplementary table of the outcome of our statistical test for growth rate recovery after bleaching, and a supplemental figure showing coral cover before and after 1998. (0.06 MB DOC) [file pone.0006324.s001.doc]

**Supplemental Information**

**Materials and Methods**

**Coral Sampling.** We collected cores using SCUBA and a hand-held reversible air drill (Ingersoll-Rand 7803RA, 500 rpm) driven by a gasoline-powered air compressor (Westward 4B220, 10 cfm). A custom-built stainless steel core barrel 6 cm in diameter and 50 cm long fitted with a brass drill head containing carbide teeth was fashioned after the design developed by the Australian Institute for Marine Science. After core removal, pre-cast concrete plugs were inserted to prevent colonization inside the coral by boring organisms and allow coral regrowth. At each site, we collected cores from the largest heads within reach of a 100-foot-long hose. Cores were drilled vertically to obtain the clearest banding pattern along the maximum growth axis.

After collection, tissue was removed using a waterpik and the cores were rinsed in fresh water and air-dried. A ~0.86 cm thick slab from the middle of each core was cut using a water-lubricated double-bladed diamond table saw. Finally, core slabs were cleaned in de-ionized water and then air-dried.

**X-radiography.** Core slabs were x-rayed at the University of California San Diego Thornton Hospital using a Siemens Polyphos 50 with a source-to-object distance of 40 inches and a setting of 63 kilovolts at 5 milliamps/second. Digital x-ray cassettes were processed using an AGFA Musica ADC Compact Plus, and images saved in DICOM format, as well as hard copy films. We measured coral growth rates from x-rays using the program CoralXDS [1].

**Anthropogenic Stress.** To calculate an index of current anthropogenic stress at our sites, we used the average Ba/Ca from the top 5 years of 5 coral cores from each site to represent sedimentation from Prouty et al. 2008 [2]. To represent nutrient input, we calculated the average chlorophyll-a from **t**ime series of satellite-derived monthly chlorophyll-a concentrations from October 1997 to October 2007 at each site obtained from NOAA Coastwatch, using data from the Sea-viewing Wide Field-of-view Sensor [3]. The relative between-site differences in sedimentation and nutrient input should both be relatively stable over time, as century-scale timeseries of Ba/Ca from each site reveal approximately constant differences between sites [4]. Human population in Utila and Cayos Cochinos was obtained from 2001 Honduran census data. Human population at Turneffe and Sapodilla was estimated by M. Mcfield based on local knowledge. While all populations have likely increased over the past century, the relative ranking of human population between sites has remained the same. Fishing pressure was estimated from the relative fish population index values from the Healthy Reefs Scorecard [5]. Fishing pressure is the only measure that may not have retained the same long-term ranking between sites (for instance if fishing expands further offshore as nearby reefs are depleted); however removing this measure does not affect the relative ranking of total impact indices described below. The above four measures of anthropogenic stress: sedimentation, nutrient input, direct human impact, and fishing pressure, were log-transformed (log(X+1)), then rescaled between 0-1 (by subtracting the minimum and dividing by the range) to put them on a unitless scale for direct comparison following Halpern et al. 2008 [6]. Each impact value was then multiplied by the coral-reef impact-specific weight determined by Halpern et al. 2007 [7]. The weight for artisinal fishing from Halpern et al. 2007 [7] was used for the fishing pressure measure calculated here. All weighted impact values were then summed to calculate our Impact Index, but since the index value used for fishing was calculated using fish abundance (with higher abundance representing less fishing), we subtracted the fish abundance factor.

To compare our relative Impact Index rankings for each site with the Halpern et al. 2008 [6] global impact map, we retrieved impact factors from four cells from their map around our sites, and averaged these values. To compare our relative impact rankings to the Healthy Reefs Scorecard Integrated Reef Health Index (IHRI) [5], we calculated the average IHRI for reefs that were surveyed close to our sites. For Turneffe, we averaged surveys at 6 sites, for Cayos, 2 sites, for Utila, 3 sites, and for Sapodilla, 3 sites. The IHRI is on a reversed scale compared to the other indices, wherein lower values represent a less healthy reef in the IHRI index. Although these stress and health metrics were calculated for a specific point in time, the measures included probably don’t rapidly fluctuate (for instance, shipping traffic and runoff in the Halpern et al. dataset or coral and algae cover in the IHRI dataset).

**Calculating Thermal Stress in 1998.** Degree heating weeks (DHW) were computed from Advanced-Very-High-Resolution-Radiometer (AVHRR) 7-day composite night-time sea surface temperature data [8]. Between 10-15 grid cells (each 0.5° x 0.5°) were averaged for each site to account for gaps in records from individual grid-cells due to cloud cover. Patterns in SST changes within sites are more highly correlated than those between sites, indicating that using the average of these multiple grid cells to reconstruct DHW over this time period is justified. During several weeks at each site, cloud cover restricted the available data. During these weeks patterns of temperature change at other sites were used to predict temperature at the missing site. For instance, if temperature for a missing week was 0.3 degrees higher than the previous week at sites without cloud cover, we added 0.3 degrees to the previous week’s temperature at the site with missing data.

DHW are calculated in several steps. First, hotspots are calculated by subtracting the local mean monthly maximum climatology, calculated by NOAA [9], which was 28.9°C for all of our sites. Hotspots are defined as any measurement that exceeds this mean temperature [9]. DHW are then calculated in moving windows, by summing the previous 12 weeks of hotspots that are at least 1°C above the mean climatology [9]. Here, we calculated the maximum DHW during 1998 at all sites and also summed all of the DHW for 1998 to compute the cumulative DHW for each site. Both of these measures result in the same heat stress ranking between sites: Cayos Cochinos with the highest heat stress, followed by the Sapodilla Cayes, Utila, and Turneffe Atoll.

**Carbonate Saturation.**

Unfortunately, no long-term datasets of carbonate saturation are available for the Mesoamerican Reef. A study in summer 1961 and 1962 found that all waters across the Belizean portion of the Mesoamerica Reef were supersaturated with respect to carbonate, but that those in southern Belize were less so than in the north [10]. This pattern is probably related to the higher influence of freshwater in the south. This overall pattern cannot explain why corals at Cayos Cochinos (also presumably experiencing similar levels of carbonate saturation as Sapodilla) recovered, while those at Sapodilla did not.

**Coral Cover Before and After 1998.** Percent coral cover pre-and post-1998 from Sapodilla, Turneffe, and Utila were compiled [11-13] and benthic point intercept transect surveys completed by JC and SW in October, 2005 using the Mesoamerican Barrier Reef System Synoptic Monitoring Program methods [14] (Fig. S1).

# References

1 Helmle KP, Kohler KE, Dodge RE (2002) Coral X-radiography Densitometry System. <http://www.nova.edu/ocean/coralxds/index.html>.

2 Prouty N, Hughen KA, Carilli J (2008) Geochemical signature of land-based activities in Caribbean coral surface samples. Coral Reefs 27: 727-742.

3 O'Reilly JE, Maritorena S, Mitchell BG, Siegel DA, Carder KL, et al. (1998) Ocean color chlorophyll algorithms for SeaWiFS. J Geophys Res 103: 24937-24953.

4 Carilli JE, Prouty NG, Hughen KA, Norris RD (in review, Mar Poll Bull) Century-scale records of land-based activities recorded in Mesoamerican coral cores.

5 Healthy Reefs Initiative (2008). Eco-health Report Card for the Mesoamerican Reef. An Evaluation of Ecosystem Health. http://www.healthyreefs.org.

6 Halpern BS, Walbridge S, Selkoe KA, Kappel CV, Micheli F, et al. (2008) A global map of human impact on marine ecosystems. Science 319: 948-952.

7 Halpern B, Selkoe KA, Michel F, Kappel CV (2007) Evaluating and ranking the vulnerability of global marine ecosystems to anthropogenic threats. Cons Bio 21: 1301-1315.

8 Li X, Pichel W, Maturi E, Clemente-Colón P, Sapper J (2001) Deriving the operational nonlinear multi-channel sea surface temperature algorithm coefficients for NOAA-15 AVHRR/3. Int J Remote Sens 22: 699-704.

9 Strong A, Barrientos CS, Duda C, Sapper J (1997) Improved satellite techniques for monitoring coral reef bleaching. Proc 8th Int Coral Reef Symp 2: 1495-1498.

10 Purdy E, Pusey WC III, Wantland KF (1975) Continental shelf of Belize--regional shelf attributes. In: Wantland KF, Pusey WC III, editors. Belize Shelf--Carbonate Sediments, Clastic Sediments, and Ecology. Tulsa, Oklahoma: American Association of Petroleum Geologists.

10 McField MD (2000) Influence of disturbance on coral reef community structure in Belize. Proc 9^th^ Int Coral Reef Symp 1: 62-68.

11 Afzal DC, Harborne AR, Raines PS “Summary of Coral Cay Conservation's monitoring data from Utila, Honduras” (Coral Cay Conservation, 2001).

12 McField M, Bood N, Fonseca A, Arrivillaga A, Franquesa Rinos A, et al. (2005) Status of the Mesoamerican Reef after the 2005 coral bleaching event. In: Wilkinson C, Souter D, editors. Status of Caribbean coral reefs after bleaching and hurricanes in 2005. Townsville: Global Coral Reef Monitoring Network. Chapter 5.

13 Almada-Villela P, Sale PF, Gold-Bouchot G, Kjerfve B (2003) Manual Of Methods For The MBRS Synoptic Monitoring Program: Selected Methods for Monitoring Physical and Biological Parameters for Use in the Mesoamerican Region. Belize City: Mesoamerican Barrier Reef Systems Project.

**Figure S1. Coral cover pre- and post-1998.** Pre-1998 data from Turneffe and Sapodilla from [10] and from Utila from [11]. No pre-1998 data are available from Cayos Cochinos. Post-1998 data from Turneffe and Sapodilla from surveys by JC and SW, October 2005. Post-1998 data from Utila from [11] and from Cayos Cochinos from [12]. Error bars represent standard error.
