# Supplementary figures and images for: Local Stressors Reduce Coral Resilience to Bleaching
Source: PLoS One. 2009 Jul 22;4(7):e6324. doi: 10.1371/journal.pone.0006324 (PMC2708352; doi:10.1371/journal.pone.0006324)

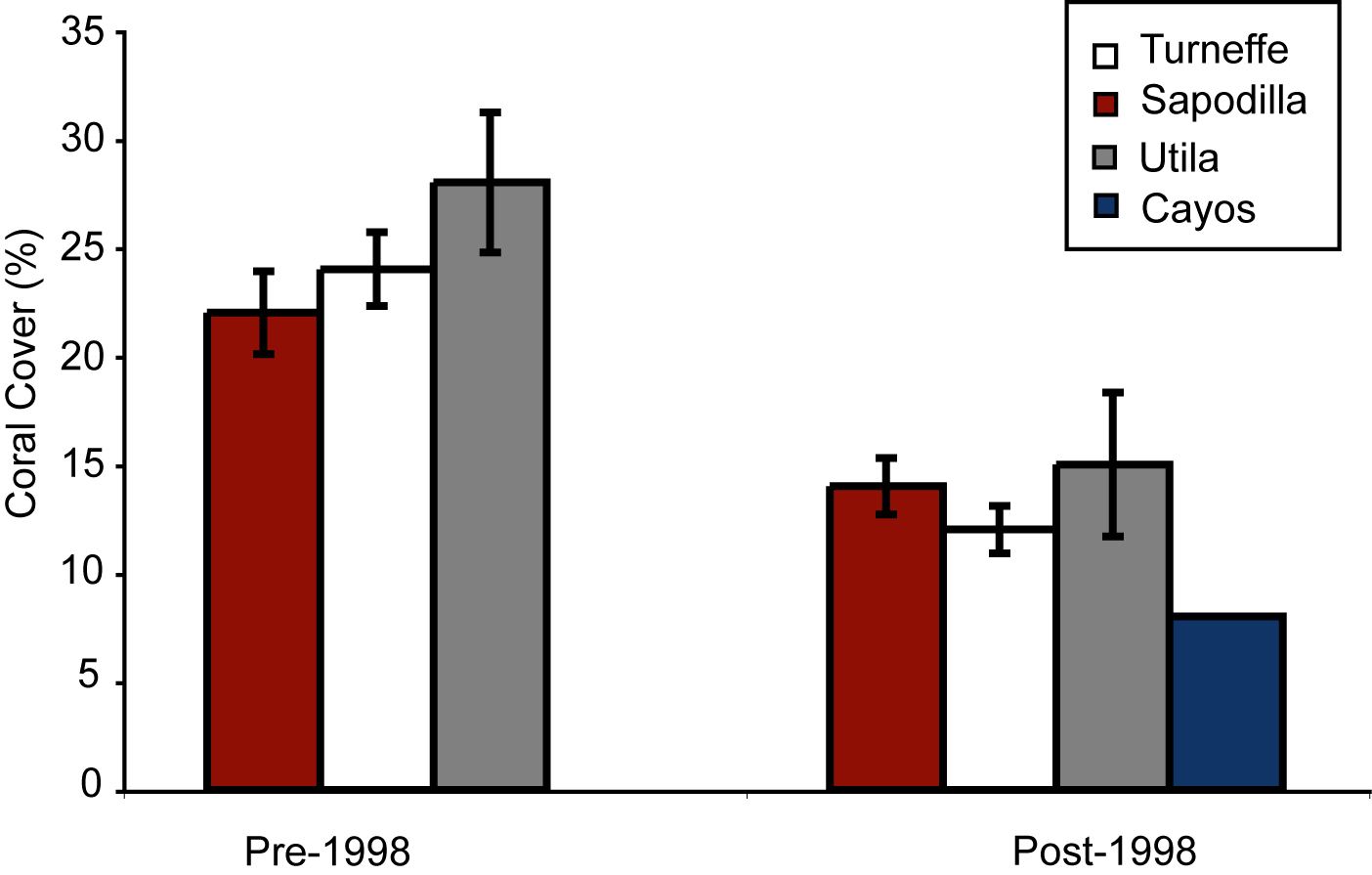

Supplement: Figure S1 — Supplemental figure of coral cover before and after 1998. (3.79 MB TIF) [file pone.0006324.s002.tif]
