# Supplementary material for: Local Stressors Reduce Coral Resilience to Bleaching
Source: PLoS One. 2009 Jul 22;4(7):e6324. doi: 10.1371/journal.pone.0006324 (PMC2708352; doi:10.1371/journal.pone.0006324)
Supplement: Table S1 — Recovery of extension rates after 1998. (0.03 MB DOC) [file pone.0006324.s003.doc]

# Table S1. Recovery of extension rates after 1998.

|  | **High Local Stress Sites** | | **Low Local Stress Sites** | |
| --- | --- | --- | --- | --- |
| **Predictor** | **Sapodilla Cayes** | **Utila** | **Turneffe Atoll** | **Cayos Cochinos** |
| Post 1998 | -0.147** | -0.281*** | NS | NS |
| Year | -0.002* | NS | NS | NS |
| Constant | 5.200** | NS | NS | NS |
| N | 53 | 53 | 53 | 53 |
| F | 20.70 | 18.26 | 2.83 | 2.19 |
| P | 0.000 | 0.000 | NS | NS |
| R^2^ | 0.45 | 0.42 | 0.10 | 0.18 |

Results of a generalized linear model estimate for changes in average extension rates after 1998, controlling for trends across years. Extension rates from 1950-1997 and 2002-2006 were compared. Significance levels are: not significant (NS) >0.05, *<0.05, **<0.01, ***<0.001.
